# Changes in myoplasmic Ca^2+^ during fatigue differ between FDB fibers, between glibenclamide-exposed and Kir6.2^-/-^ fibers and are further modulated by verapamil

**DOI:** 10.14814/phy2.12303

**Published:** 2015-03-05

**Authors:** David Selvin, Jean-Marc Renaud

**Affiliations:** Department of Cellular and Molecular Medicine, University of OttawaOttawa, Ontario, Canada

**Keywords:** Ca^2+^ channel, calcium, fatigue, Kir6.2 knockout

## Abstract

One objective of this study was to document how individual FDB muscle fibers depend on the myoprotection of KATP channels during fatigue. Verapamil, a CaV1.1 channel blocker, prevents large increases in unstimulated force during fatigue in KATP-channel-deficient muscles. A second objective was to determine if verapamil reduces unstimulated [Ca^2+^]i in KATP-channel-deficient fibers. We measured changes in myoplasmic [Ca^2+^] ([Ca^2+^]i) using two KATP-channel-deficient models: (1) a pharmacological approach exposing fibers to glibenclamide, a channel blocker, and (2) a genetic approach using fibers from null mice for the Kir6.2 gene. Fatigue was elicited with one tetanic contraction every sec for 3 min. For all conditions, large differences in fatigue kinetics were observed from fibers which had greater tetanic [Ca^2+^]i at the end than at the beginning of fatigue to fibers which eventually completely failed to release Ca^2+^ upon stimulation. Compared to control conditions, KATP-channel-deficient fibers had a greater proportion of fiber with large decreases in tetanic [Ca^2+^]i, fade and complete failure to release Ca^2+^ upon stimulation. There was, however, a group of KATP-channel-deficient fibers that had similar fatigue kinetics to those of the most fatigue-resistant control fibers. For the first time, differences in fatigue kinetics were observed between Kir6.2^-/-^ and glibenclamide-exposed muscle fibers. Verapamil significantly reduced unstimulated and tetanic [Ca^2+^]i. It is concluded that not all fibers are dependent on the myoprotection of KATP channels and that the decrease in unstimulated force by verapamil reported in a previous studies in glibenclamide-exposed fibers is due to a reduction in Ca^2+^ load by reducing Ca^2+^ influx through CaV1.1 channels between and during contractions.

## Introduction

Muscle fatigue is defined as a transient and recoverable decline in muscle force or power with repeated or continuous muscle contractions and is a protective mechanism to preserve cellular integrity via regulation of myoplasmic Ca^2+^ concentration ([Ca^2+^]i) and ATP levels (McKenna et al. 2008). Such regulation is important because chronic elevation of [Ca^2+^]i and/or large decrease in ATP levels are known to cause fiber damage, even cell death. It implies that muscle fibers must have mechanisms that (1) detect metabolic stress or Ca^2+^ and (2) decrease contraction to reduce ATP hydrolysis and Ca^2+^ levels. The ATP-sensitive K^+^ (KATP) channel is a component of these mechanisms. The channel links energy metabolism to the electrical activity of the cell membrane as it is activated during metabolic stress when intracellular ATP and pH decrease and intracellular ADP and extracellular adenosine increase (Noma 1983; Vivaudou et al. 1991; Standen et al. 1992). More importantly, there is now evidence for the activation of the channel during metabolic stress (Pedersen et al. 2009a,b).

A lack of KATP channel activity under low-intensity workloads such as one Hz twitch contractions for 5–7 min prevent the hyperpolarization of the resting membrane potential and reduction in action potential amplitude normally observed in wild-type muscle (Zhu et al. 2014). During high intensity workloads leading to fatigue, however, a lack of KATP channel activity results in fiber damage in both cardiac and skeletal muscles during exercise (Zingman et al. 2002; Kane et al. 2004; Thabet et al. 2005). During fatigue*,* KATP channel deficiency also leads to contractile dysfunctions, defined by Cifelli et al. (2007), as any event from the generation of action potentials to the actin–myosin interaction that is depressed in a manner not associated with the normal process of fatigue, and that eventually incapacitates muscle from generating force. Interestingly, one fatigue bout acutely increases fatigue resistance and lowers muscle dependency on the myoprotective effects of KATP channels, a phenomenon known as fatigue preconditioning (Boudreault et al. 2010). However, fatigue preconditioning only last three hrs; so, following any long period of muscle inactivity, KATP channels remain important for myoprotection.

There are two mechanisms by which the KATP channel prevents fiber damage. The first mechanism involves lower action potential amplitude (Matar et al. 2000; Gong et al. 2003; Zhu et al. 2014) that reduces Ca^2+^ release from the sarcoplasmic reticulum and eventually force generation at the sarcomere level (Zhu et al. 2014). The net effect is a decrease in Ca^2+^ ATPase activity as less Ca^2+^ must be pumped back in the sarcoplasmic reticulum and a decrease in myosin ATPase activity because of less actomyosin links. The second mechanism is the maintenance of the resting membrane potential. During fatigue or metabolic inhibition, the resting membrane potential depolarizes by about 10–15 mV, but in the absence of KATP channels it can depolarize by more than 40–50 mV (Gramolini and Renaud 1997; Cifelli et al. 2008). Concomitant with the large membrane depolarization are, in the absence of any stimulation, large increases in [Ca^2+^]i and force, here referred to as unstimulated [Ca^2+^]i and force, respectively. The large increase in unstimulated [Ca^2+^]i and force then causes increases in ATP demand worsening the metabolic stress associated with fatigue, whereas the elevated [Ca^2+^]i may also be the cause of fiber damage (Jackson et al. 1984; Gissel and Clausen 2000).

When glycolytic EDL muscle fibers are stimulated repetitively, membrane conductance first decreases due to a reduced activity of ClC-1 Cl^−^ channels; eventually after ∽2000 action potentials membrane conductance increases due to the activation of ClC-1 and KATP channels when a metabolic stress occurs (Pedersen et al. 2009a). However, the increase in membrane conductance could not be observed in the oxidative soleus muscle fibers even after 15,000 action potentials possibly because of a lack of metabolic stress (Pedersen et al. 2009b). Furthermore, t-tubular KATP channel protein content in skeletal muscle fibers depends on either the metabolic profile and/or the incidence of metabolic stress with the greatest channel content in the most glycolytic fibers and the lowest content in the most oxidative fibers; that is, in the order of type IIB > IIX > IIA > I fibers (Banas et al. 2011). What remains to be determined is the response of different fibers to a lack of KATP channel activity. So, one objective of this study was to document how the absence of KATP channel activity adversely affects the regulation of myoplasmic Ca^2+^ during fatigue among fibers. Mouse FDB was chosen for this study because several viable and contracting fibers can be obtained following a collagenase digestion (Selvin et al. 2014) and because it is composed of a spectrum of type I-IIA (6%), I-IIX (8%), IIA (19%); IIA-IIX (32%); IIX (21%), with no fiber expressing IIB myosin (Banas et al. 2011).

Exposing KATP-channel-deficient FDB muscle to lower extracellular [Ca^2+^] or submaximal concentration of verapamil, a CaV1.1 or L-type Ca^2+^ channel blocker, prevents the large increase in unstimulated force, reduces fatigue rate, and improves force recovery following fatigue (Cifelli et al. 2008). Based on these results, Cifelli et al. suggested (1) that the large unstimulated force involves Ca^2+^ influx through CaV1.1 channels that remain open between contractions because of the cell membrane depolarization to −30 mV allowing for large increase in unstimulated [Ca^2+^]i and (2) that the slower fatigue rate and better recovery in the presence of verapamil was due to less Ca^2+^-induced fiber damage. [Ca^2+^]i was, however, not measured in the Cifelli et al. study. So, it is possible that the lower unstimulated [Ca^2+^]i is not just the results of lower Ca^2+^ influx through CaV1.1, but also to lower Ca^2+^ release during contractions since the maintenance of the plateau phase during a tetanus requires a Ca^2+^ influx through CaV1.1 channels (Cifelli et al. 2008). So, the second objective of this study was to determine how verapamil affects unstimulated and tetanic [Ca^2+^]i in various fiber types that have no KATP channel activity.

## Methods

### Animals and K_ATP_-channel-defficient muscle fibers

Experiments were carried out using single fibers from flexor digitorum brevis (FDB) muscles. K_ATP_-channel-deficient muscle fibers were obtained by (1) exposing fibers from CD-1 mice (Charles River, Canada) to glibenclamide, a K_ATP_ channel inhibitor (pharmacological model) and (2) using Kir6.2^-/-^ fibers (provided by Dr. S Seino, University of Kobe, Japan), a genetic model that does not express functional K_ATP_ channels in skeletal muscle (Miki et al., 2002). Mice were 2–4 months in age and weighed 20–30 g. Mice were fed ad libitum, and housed according to the guidelines of the Canadian Council for Animal Care (CCAC). The Animal Care Committee of the University of Ottawa approved all experimental procedures used in this study. Mice were anesthetized with a single intraperitoneal injection of 2.2 mg ketamine, 0.44 mg xylazine and 0.22 mg acepromazine per 10 g of body mass. Subjects were then killed by cervical dislocation.

### Single fiber preparation

Single fibers were isolated from FDB muscles as described by Selvin et al. (2014). Briefly, FDB muscles were incubated 3 h at 37°C in minimum essential medium with Earle's salt and L-glutamine (MEM, Gibco, Burlington, ON, Canada) containing 0.2% (w/v) collagenase type I (Worthington, Lakewood, NJ), and supplemented with 10% (v/v) fetal bovine serum (FBS, Gibco, heat inactivated) to prevent fiber supercontraction, 100 units/mL of penicillin, and 100 μg/mL of streptomycin (Gibco). Fibers were separated by gentle trituration in 3 mL collagenase-free MEM. One hundred microliter of concentrated fiber-containing medium was placed on a Matrigel (VWR, Canada) precoated 12-mm-diameter coverslip (VWR, Edmonton, AB, Canada). Fibers were incubated another 30 min to allow for fibers to settle on the Matrigel and become fixed. Culture medium was then added to cover the entire coverslip and fibers were incubated for at least another 30 min before being used for Ca^2+^ measurements. All measurements were carried out the day the fibers were prepared because the stimulation threshold and the number of contracting fibers remained constant for at least 3 h after being transferred to the experimental chamber used to measure [Ca^2+^]i, while after an overnight incubation at 37°C some fibers completely lose their capacity to contract, whereas others have an increased stimulation threshold over a three hr period in the experimental chamber (Selvin et al. 2014).

### Experimental setup & solutions

Coverslips containing single FDB fibers were mounted into a 370 μL chamber (model RC-25, Warner Instruments, Hamden, CT). Fibers were continuously perfused in physiological solution with a flow rate of 5 mL/min. Experimental temperature of 37°C was controlled for by simultaneously heating the plate in which the chamber was mounted, and the physiological solution running through the chamber, using a dual channel heater controller (model TC-344B, Warner Instruments). The fiber temperature was about 22°C after positioning the RC-25 chamber on the microscope, and that temperature had to be increased to a the physiological 37°C. As discussed by Selvin et al. (2014), the temperature was increased from 22°C to 37°C at a rate of 2°C every 100 sec to prevent loss of fiber contractility.

The control physiological solution contained (mmol/L): 118.5 NaCl, 4.7 KCl, 2.4 CaCl_2_, 3.1 MgCl_2_, 25 NaHCO_3_, 2 NaH_2_PO_4_, 5.5 D-glucose and 0.2% FBS. All solutions were continuously bubbled with 95% O_2_–5% CO_2_ and had a pH of 7.4. FBS was added in all solutions because it prevents (1) a complete loss of contractility and (2) increases in stimulation threshold over time especially when fibers are stressed with a fatigue bout (Selvin et al. 2014). Glibenclamide-containing solution (10 μmol/L) was obtained by first dissolving glibenclamide in DMSO and adding it to the physiological solution. Solutions containing verapamil (1 μmol/L) were obtained by adding verapamil directly to the saline solution, as this compound is water soluble. In all experiments DMSO concentration was kept at 0.1% (v/v).

### Fiber stimulation

Fibers were stimulated using field stimulation, generated by two platinum electrodes running along each side of the chamber containing the fibers. The electrodes were connected to a Grass S88 stimulator and SIU5 isolation unit (Grass Technologies, West Warwich, RI) which were used to generate 200 msec trains of 0.3 msec 10 V pulses at a frequency of 140 Hz.

### [Ca^2+^]_i_ measurement

Fibers were loaded with Fura2 by incubating fibers 30 min at 37°C in culture medium containing 5 μmol/L Fura-2 AM (Molecular Probes, Burlington, ON, Canada), similar to the protocol described by Westerblad and Allen (1991). Fura-2 was alternatively excited at wavelengths of 340 and 380 nm, and light emission was measured at 505 nm. Alternating between the different excitation wavelengths and fluorescence measurement were carried out using the IonOptix dual fluorescent contractility device (IonOptix, Milton, MA) employing the following filters: 340 ± 12 nm, 380 ± 6 nm, and 505 ± 6 nm. Data acquisition was set at 200 Hz; the 505 nm light emitted in 2 msec during excitation at 340 nm was divided by the 505 nm light emitted in 2 msec during excitation at 380 nm. [Ca^2+^]i was then calculated from the ratio as previously described (Boudreault et al. 2010) using the following equation: 

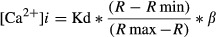
 where,

Kd is the dissociation constant of fura-2 for Ca^2+^ (37°C: 224 nmol/L, (Li et al. 2008), RMIN being 89 ± 0.7% of the ratio measured in resting fibers, RMAX being 126.1 ± 7.5% of the ratio during a tetanic contraction under control conditions, and β the fluorescence at 380 nm excitation of Ca^2+^-free divided by Ca^2+^-bound Fura-2, being 3.17 ± 0.72 (Boudreault et al. 2010). Measurements of RMIN was carried out by exposing fibers to a physiological solution containing no Ca^2+^, 1 mmol/L EGTA, a Ca^2+^ buffer, and ionomycin, a Ca^2+^ ionophore, in order to reduce [Ca^2+^]i to the lowest possible level. RMAX was measured by exposing fibers to a solution containing 10 μmol/L Ca^2+^ and ionomycin. As described by Westerblad and Allen (1991), RMAX measurements are complicated by strong contractions and changes in fura-2 fluorescence characteristics, so it cannot be successfully measured in all fibers. So, as described earlier (Westerblad and Allen 1991), average values were used for RMIN and RMAX. For this study, we present [Ca^2+^]i and not ratio values because (1) our mean tetanic [Ca^2+^]i values reported in this study are within the range reported in previous studies in which fura-2 or indo-1 was used (Westerblad and Allen 1991; Chin and Allen 1996, 1998; Chin et al. 1997) and (2) our analyses involved the subtraction of tetanic [Ca^2+^]i at the end of fatigue from the prefatigue value and such calculations cannot be performed using ratio values because the [Ca^2+^]i – ratio relationship is nonlinear.

Fura-2 fluorescence is being lost over time due to either photobleaching or leak through cell membrane, the latter being much greater at 37°C than at 25°C (Bourassa 2006). Probenecid, which can reduce the leak, significantly alters fatigue kinetics at 37°C (F. Bourassa and J. M. Renaud, unpubl. data). However, both unstimulated and tetanic ratios remain constant over one hr despite a 80% decrease in the fluorescence measured with an excitation at 380 nm while fibers were at rest (Bourassa 2006). For this study, all experiments were completed within 1 h for each fiber.

### Statistical analysis

For most of the experiments, ANOVAs were used to determine significant differences. Split plot designs were used because fibers were tested at all-time levels. ANOVA calculations were made using the Version 9.3 GLM (General Linear Model) procedures of the Statistical Analysis Software (SAS Institute Inc., Cary, NC). When a main effect or an interaction was significant, the least square difference (L.S.D.) was used to locate the significant differences (Steel and Torrie 1980). The Fishers Exact test was used when proportions of fibers were calculated (Figs. 2A, 4). For each variable, a 2 × 4 test was first carried out, the four categories being control, glibenclamide, glibenclamide-verapamil and Kir6.2^-/-^ fibers. If the model gave a significant probability, then individual 2 × 2 tests were carried out to determine significant differences versus control, glibenclamide versus glibenclamide-verapamil, and glibenclamide versus Kir6.2^-/-^. The word “significant” refers only to a statistical difference (*P *< 0.05).

## Results

**Figure 1:**
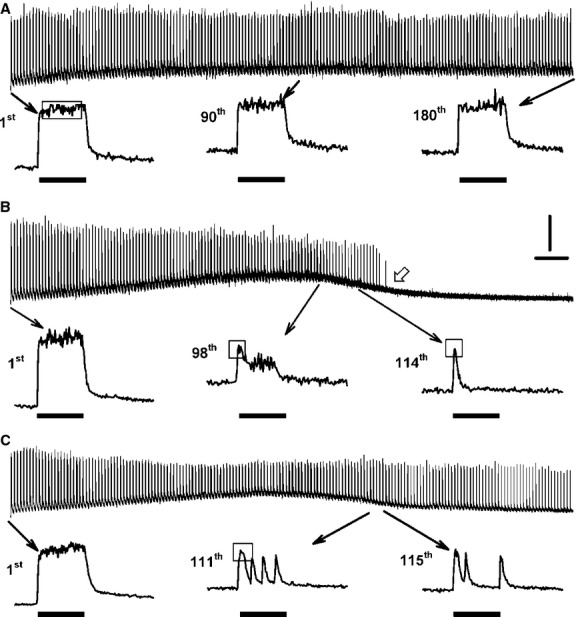
Examples of Ca^2+^ traces showing complete failure to release Ca^2+^, partial and complete fade. Traces were from (A) control wild-type fiber; (B) 10 μmol/L glibenclamide-exposed wild-type fiber; (C) Kir6.2^-/-^ fiber. Fatigue was elicited with one contraction every sec for 3 min. The 1st, 90th, and 180th contractions in A are examples for which a plateau phase is maintained during a tetanic contraction. The white arrow at 121 sec in B demonstrates a complete failure to release Ca^2+^ upon stimulation. The 98th contraction in B, the 111th and 115th in C are examples of partial fade. The 114th contraction in B is an example of complete fade. Vertical line: 0.5 μmol/L Ca^2+^; horizontal line: 10 sec; horizontal black bars: 200 msec long train of pulses at 140 Hz; open horizontal bars showed how maximum tetanic [Ca^2+^]i was calculated under various fade conditions (see text for more details).

In unfatigued muscle fibers, [Ca^2+^]i rapidly increased upon stimulation maintaining a constant [Ca^2+^]i or plateau phase until the end of the stimulation such as the first contraction in Figure1A. Changes in the shape of the plateau phase were observed when fibers were fatigued with one contraction every seconds for 3 min. As shown in Figure1A, some fibers maintained the plateau phase throughout the fatigue period. Other fibers failed to maintain a plateau phase. It usually started with partial fade. This situation was observed when after reaching a peak, [Ca^2+^]i decreased to a lower level (98th contraction in Fig.1B) or appeared as a series of peaks (111th and 115th contractions in Fig.1C). Complete fade occurred when prior to the end of the tetanic stimulation [Ca^2+^]i returned back to the unstimulated [Ca^2+^]i level (114th contraction in Fig.1B). Finally, some fibers completely failed to release Ca^2+^ upon stimulation before the end of the fatigue period (Fig.1B).

**Figure 2:**
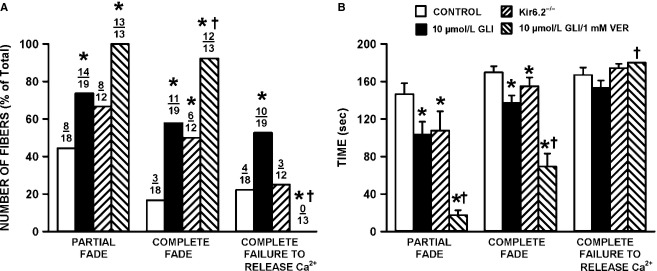
The lack of KATP channel activity significantly increased the number of fibers with partial and complete fade. (A) Number of fibers with partial, complete fade and complete failure to release Ca^2+^ are as defined in Figure1. Numbers at the top of each bar represent the number of fibers with fade or failure over total number of tested fibers. (B) Mean times that fibers were able to maintain a Ca^2+^ plateau phase while being stimulated. For complete failure, partial and complete fade, we recorded the first time it occurred. In the absence of complete failure, partial or complete fade, a value of 180 sec was recorded. Statistical test in A was the Fishers Exact test and in B an ANOVA and L.S.D. using a probability of *P* < 0.05. *Proportions of fibers in A or mean time value in B were significantly different from the mean time value of control fibers, ^†^Proportions of fibers in A or mean time value in B were significantly different from the mean time value for glibenclamide-exposed fibers.

Partial fade was observed in 44% of the control fibers (Fig.2A). This proportion significantly increased to 74% in glibenclamide-exposed wild-type fibers. The proportion was also higher in Kir6.2^-/-^ fibers, being 67%, but the difference did not reach significance. The proportion of fibers with partial fade increased to 100% for those exposed to both 10 μmol/L glibenclamide and 1 μmol/L verapamil, a CaV1.1 channel blocker. For three control fibers, partial fade became a complete fade, whereas for four of them partial fade eventually lead to a complete failure to release Ca^2+^ upon stimulation. Compared to control fibers (17%), complete fade was significantly greater in the presence of glibenclamide (58%), of glibenclamide/verapamil (92%), and in Kir6.2^-/-^ fibers (50%). Complete failure to release Ca^2+^ was observed in 22% of control fibers, a value significantly different from the 53% for glibenclamide-exposed fibers and 0% in glibenclamide/verapamil-exposed fibers. The proportion of Kir6.2^-/-^ fibers (25%) with complete Ca^2+^ failure was similar to control.

Next, we measured how long fibers were able to maintain a plateau phase (Fig.2B). Control fibers maintained a constant plateau phase during stimulation for up to a mean of 146 sec (equivalent to 146 contractions) before partial fade was observed in some fibers. Mean time to partial fade was significantly shorter for Kir6.2^-/-^ and glibenclamide-exposed fibers, with values of 108 and 103 sec, respectively. Partial fade eventually became complete fade by a mean time of 170 sec for control fibers, a value that was significantly longer than the 137 and 155 sec for Kir6.2^-/-^ and glibenclamide-exposed fibers, respectively. Complete failure to release Ca^2+^ occurred at 167, 153, and 174 sec for control, glibenclamide-exposed and Kir6.2^-/-^ fibers, respectively. Glibenclamide- and verapamil-exposed fibers had the shortest time period before partial fade occurred (18 sec). However, of the total of 29 fibers exposed for 30 min to glibenclamide prior to fatigue, only three (10%) had some partial fade compared to 9 of 14 (64%) fibers concomitantly exposed to glibenclamide and verapamil. That is, a large portion of glibenclamide- and verapamil-exposed fibers already had partial fade prior to the fatigue stimulation. Notably, complete fade started within 69 sec of fatiguing stimulation, but complete failure to release Ca^2+^ did not occur for the glibenclamide- and verapamil-exposed fibers.

### Changes in peak and plateau tetanic [Ca^2+^]i during fatigue

Normally, several [Ca^2+^]i data points are averaged to calculate [Ca^2+^]i values because of the signal noises associated with light measurement. During contractions, the averaging is easy when there is a [Ca^2+^]i plateau phase as shown in Figure1A, but not when fade occurred. To best illustrate how tetanic [Ca^2+^]i during contractions changed during fatigue, we measured two parameters. The first parameter was the plateau tetanic [Ca^2+^]i to represent the average [Ca^2+^]i during a tetanic contraction. In unfatigued fibers, [Ca^2+^]i reached its maximum in <20 msec following the first stimulation (usually within 8 or 12 msec). So, plateau tetanic [Ca^2+^]i was calculated by averaging all [Ca^2+^]i data points from the 20th to the 200th msec (the time of the last stimulation). The second parameter was the peak tetanic [Ca^2+^]i. When there was no fade, peak tetanic [Ca^2+^]i was calculated from the 20th to the 200th msec as shown in Figure1A; so, in the absence of any fade peak and plateau tetanic [Ca^2+^]i had the same value. When there was a fade, such as the 98th contractions in Figure1B, an average [Ca^2+^]i value was calculated from at least 10 data points; in other cases, such as the 114th contractions in Figure1B, the peak was so sharp that peak [Ca^2+^]i could only be calculated by averaging three data points. It is important to note that for the latter case, peak [Ca^2+^]i was underestimated as the Ca^2+^ fura-2 interaction is too slow for fura-2 to fully follow rapid changes in [Ca^2+^]i (Liu et al. 1997). So for this study, the peak [Ca^2+^]i was not used to estimate the maximum [Ca^2+^]i achieved during tetanic contractions, but to better illustrate the occurrence of fade, being indicated when the plateau [Ca^2+^]i was smaller than the peak [Ca^2+^]i, the largest differences between the two parameters being observed for the glibenclamide- and verapamil-exposed fibers (Fig. 7).

**Figure 3:**
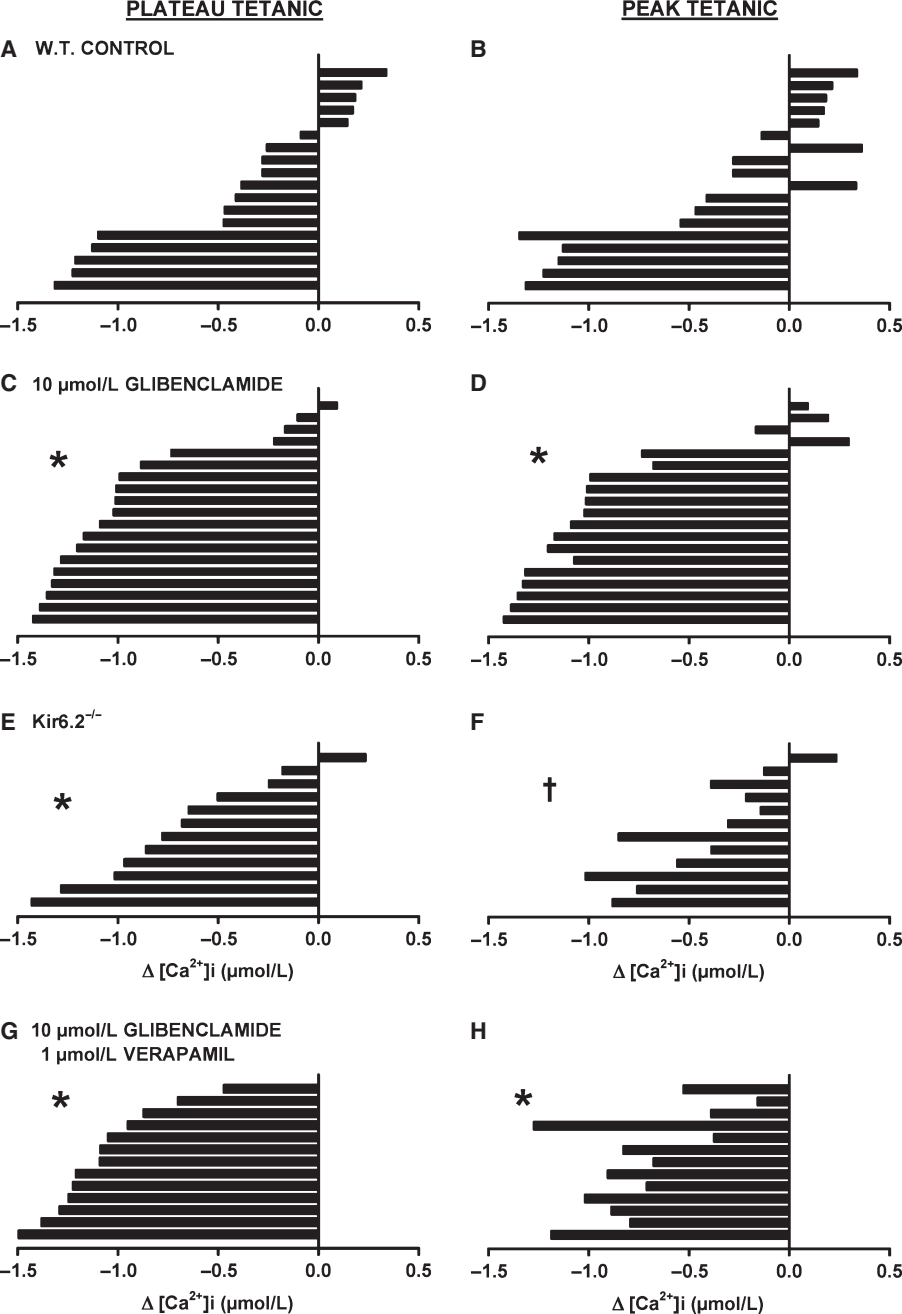
The lack of KATP channel activity increased the number of fibers with large decreases in tetanic [Ca^2+^]i. Changes in plateau (A, C, E, G) and in peak (B, D, F, H) tetanic [Ca^2+^]i were calculated as the difference in plateau or peak [Ca^2+^]i between 0 and 180 sec; a negative value indicates a decreased [Ca^2+^]i. Each horizontal bar represents a different fiber in the order from the highest increase to the largest decrease in plateau tetanic [Ca^2+^]i. The same order was maintained for peak tetanic [Ca^2+^]i. (A, B) wild-type control, (C, D) 10 μmol/L glibenclamide-exposed, (E, F) Kir6.2^-/-^ and (G, H) 10 μmol/L glibenclamide- and 1 μmol/L verapamil-exposed fibers. *Indicate that the proportion of fibers with a decrease in tetanic [Ca^2+^]i >0.5 μmol/L was significantly different than the proportion for control fibers, ^†^Indicate that the proportion of fibers with a decrease in tetanic [Ca^2+^]i >0.5 μmol/L was significantly different from the glibenclamide-exposed fibers, Fishers Exact test, *P* < 0.05.

To illustrate how peak and plateau [Ca^2+^]i changed during fatigue between fibers, we first calculated the differences in plateau and peak tetanic [Ca^2+^]i between the first and last contractions of the fatigue period. For wild-type control, five fibers had greater plateau tetanic [Ca^2+^]i at the end than at the beginning of fatigue (Fig.3A). Another eight fibers had decreases in plateau tetanic [Ca^2+^]i ranging from 0.09 to 0.47 μmol/L. Interestingly, two of these fibers had greater peak tetanic [Ca^2+^]i at the end than at the beginning of fatigue (Fig.3B); the situation occurred because the two fibers had significant fade resulting in a net decrease in plateau [Ca^2+^]i. The last five fibers had decreases in peak and plateau tetanic [Ca^2+^]i exceeding 1.10 μmol/L.

**Figure 4:**
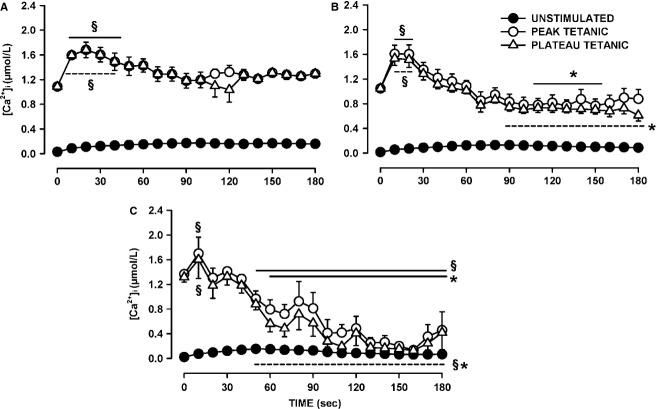
Changes in unstimulated, peak, and plateau tetanic [Ca^2+^]i during fatigue among wild-type control fibers. Fibers were separated into three groups: (A) fibers for which plateau tetanic [Ca^2+^]i at the end of fatigue was greater than the prefatigue value (*n* = 5); (B) fibers for which the final decreases in plateau tetanic [Ca^2+^]i were <0.5 μmol/L (*n* = 8); (C) fibers for which the decreases exceeded 0.5 μmol/L (*n* = 5). See Figure8 for a better resolution in unstimulated [Ca^2+^]i between fiber groups. ^§^Mean peak (solid line) or plateau (dashed line) tetanic [Ca^2+^]i was significantly different from the prefatigue mean value (Time 0 sec). *Mean peak or plateau tetanic [Ca^2+^]i was significantly different from the mean value for the fibers in A, ANOVA, and L.S.D. *P* < 0.05.

The main effect of abolishing KATP channel activity with glibenclamide was a significant increase in the proportion of fibers with decreases in both peak and plateau tetanic [Ca^2+^]i exceeding 0.5 μmol/L (Fig.3C and D). Only one fiber had a greater plateau tetanic [Ca^2+^]i at the end of fatigue and another three fibers had small decreases that were <0.22 μmol/L. Of those four fibers, three had higher peak tetanic [Ca^2+^]i at the end of fatigue. For Kir6.2^-/-^ fibers, the proportion of fibers with decreases exceeding 0.5 μmol/L was not significantly different from control fibers for peak [Ca^2+^]i while it was for plateau [Ca^2+^]i (Fig.3E and F), the reason being that fade was a main factor in Kir6.2^-/-^ fibers toward the end of the fatigue period (Fig.6). None of the glibenclamide- and verapamil-exposed fibers had greater peak or plateau tetanic [Ca^2+^]i at the end than at the beginning of fatigue (Fig.4G and H), and the proportion of fibers with decreases in peak and plateau tetanic [Ca^2+^]i >0.5 μmol/L was significantly larger than for control fibers, but not different from fibers exposed to glibenclamide alone.

To better understand the difference in fatigue kinetics between fibers, and in light of the distinct groupings that became apparent while elucidating the variation in plateau and peak [Ca^2+^]i among fibers, we also plotted the change in tetanic [Ca^2+^]i over time during fatigue separating wild-type control fibers into three groups: (1) those which had a higher plateau tetanic [Ca^2+^]i at the end of fatigue; (2) fibers with decreases ranging between 0.09 and 0.47 μmol/L; and (3) fibers with decreases exceeding 0.5 μmol/L. For the first group, plateau tetanic [Ca^2+^]i significantly increased from 1.2 to 1.7 μmol/L and then returned to values close to prefatigue values within 90 sec (Fig.4A). Peak and plateau tetanic [Ca^2+^]i were similar because of a very low incidence of fade for this group. For the second group (Fig.4B), the decreases in mean plateau tetanic [Ca^2+^]i was significantly greater when compared with the first group, but did not reach significance when compared with the mean prefatigue level. The largest decreases in plateau and peak tetanic [Ca^2+^]i occurred with the third group of fibers with the values becoming just above those of unstimulated [Ca^2+^]i (Fig.4C). This was because the third group had the largest number of fibers with complete failure to release Ca^2+^ upon stimulation.

**Figure 5:**
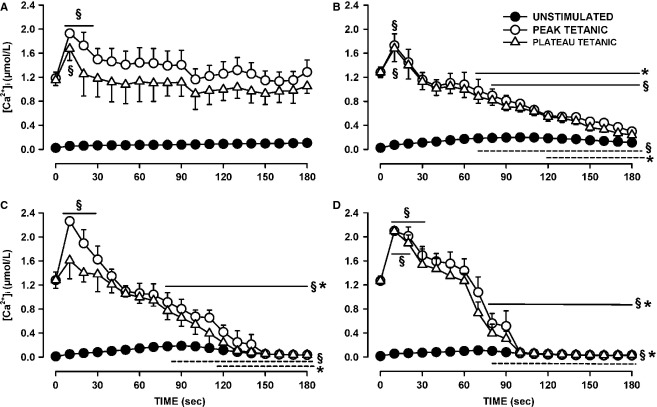
Changes in unstimulated, peak, and plateau tetanic [Ca^2+^]i during fatigue between wild-type fibers exposed to 10 μmol/L glibenclamide. Fibers were separated into four groups. (A) fibers for which at the end of fatigue plateau tetanic [Ca^2+^]i either had increased or decreased by <0.22 μmol/L (*n* = 4); (B) fibers for which decreases in plateau tetanic [Ca^2+^]i exceeded 0.5 μmol/L but released Ca^2+^ upon stimulation throughout the entire fatigue period (*n* = 7); (C) fibers with decreases in plateau tetanic [Ca^2+^]i exceeding 0.5 μmol/L and suddenly failed to release Ca^2+^ between the 120th and 150th sec (*n* = 3) or (D) between the 90th and 100th sec (*n* = 5). A lack of Ca^2+^ release can be seen when there is no difference between unstimulated and tetanic [Ca^2+^]i. See Figure8 for a better resolution in unstimulated [Ca^2+^]i between fiber groups. ^§^Mean peak (solid line) or plateau (dashed line) tetanic [Ca^2+^]i was significantly different from the prefatigue mean value (Time 0 sec). *Mean peak or plateau tetanic [Ca^2+^]i was significantly different from the mean for the fibers in A, ANOVA and L.S.D. *P* < 0.05.

The glibenclamide-exposed fibers were divided into four groups. The first group comprised of fibers with either higher plateau tetanic [Ca^2+^]i or decreases not exceeding 0.22 μmol/L (i.e., the top four fibers in Fig.3C). These fibers had on average the same plateau tetanic [Ca^2+^]i at the end of fatigue than at the beginning (Fig.5A). The mean peak tetanic [Ca^2+^]i were higher than the mean plateau tetanic [Ca^2+^]i because for this group fade was a significant factor. The second group of fibers had decreases in plateau tetanic [Ca^2+^]i exceeding 0.5 μmol/L and were able to release Ca^2+^ upon stimulation until the end of the fatigue period (Fig.5B). The last two groups of glibenclamide-exposed fibers also had decreases in plateau tetanic [Ca^2+^]i exceeding 0.5 μmol/L, but they eventually completely failed to release Ca^2+^ upon stimulation; the two groups were differentiated by time to failure, one group by 150 sec (Fig.5C), the other by 100 sec (Fig.5D).

**Figure 6:**
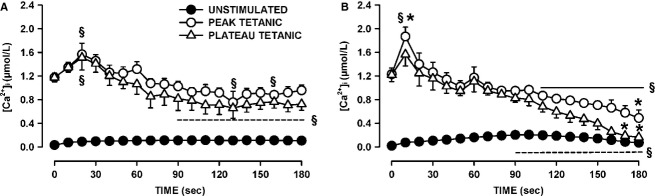
Changes in unstimulated, peak, and plateau tetanic [Ca^2+^]i during fatigue between Kir6.2^-/-^ fibers. Fibers were separated into two groups: (A) fibers for which plateau tetanic [Ca^2+^]i decreased between 0.2 and 0.5 μmol/L (*n* = 5); (B) fibers for which the decreases in plateau tetanic [Ca^2+^]i exceeded 0.5 μmol/L (*n* = 6). See Figure8 for a better resolution in unstimulated [Ca^2+^]i between fiber groups. ^§^Mean peak (solid line) or plateau (dashed line) tetanic [Ca^2+^]i was significantly different from the prefatigue mean value (Time 0 sec). *Mean peak or plateau tetanic [Ca^2+^]i was significantly different from the mean for the fibers in B, ANOVA, and L.S.D. *P* < 0.05.

Kir6.2^-/-^ fibers were divided into two groups. The first group comprised fibers with decreases in plateau tetanic [Ca^2+^]i <0.7 μmol/L. For these fibers mean plateau [Ca^2+^]i at the end of fatigue was 0.73 μmol/L, a value significantly less than the prefatigue mean of 1.18 μmol/L (Fig.6A). The second group of Kir6.2^-/-^ fibers had decreases in plateau [Ca^2+^]i exceeding 0.8 μmol/L and at the end of the fatigue period had a mean plateau [Ca^2+^]i of 0.16 μmol/L (Fig.6B), a value that was significantly less than the mean value for the first group (Fig.6A). Differences between peak and plateau [Ca^2+^]i became apparent by 50 sec for the first group and 90 sec for the second group.

**Figure 7:**
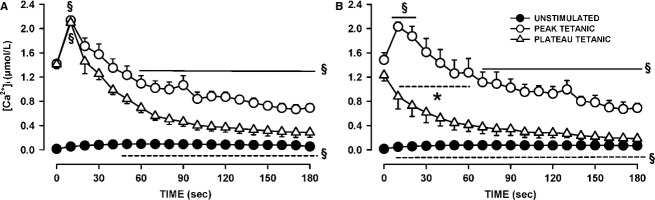
Changes in unstimulated, peak, and plateau tetanic [Ca^2+^]i during fatigue between wild-type fibers exposed to 10 μmol/L glibenclamide and 1 μmol/L verapamil. Fibers were separated into two groups: (A) fibers for which plateau tetanic [Ca^2+^]i increased during the first 10 sec (*n* = 8); (B) fibers for which plateau tetanic [Ca^2+^]i started to decreased immediately (*n* = 7). See Figure8 for a better resolution in unstimulated [Ca^2+^]i between fiber groups. ^§^Mean peak (solid line) or plateau (dashed line) tetanic [Ca^2+^]i was significantly different from the prefatigue mean value (Time 0 sec). *Mean peak or plateau tetanic [Ca^2+^]i was significantly different from the mean of the fibers in A, ANOVA, and L.S.D. *P* < 0.05.

Glibenclamide- and verapamil-exposed fibers were divided into one group with increases in plateau tetanic [Ca^2+^]i (Fig.7A), and another with no initial increases (Fig.7B). Note that for both groups, the plateau tetanic [Ca^2+^]i were much less than for the peak tetanic [Ca^2+^]i as all fibers in this group had substantial partial and complete fade, being in fact the largest of all four experimental conditions.

### Unstimulated [Ca^2+^]i

**Figure 8:**
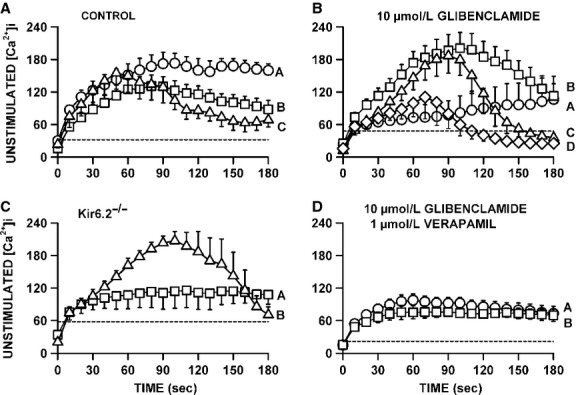
Verapamil significantly reduced the increase in unstimulated [Ca^2+^]i when KATP channels are blocked with 10 μmol/L glibenclamide. (A) Changes in unstimulated [Ca^2+^]i during fatigue for the three groups of control fibers as shown in Figure5A–C; (B) the same for glibenclamide-exposed fibers as shown in Figure6A–D, (C) for Kir6.2^-/-^ fibers in Figure7A–C and (D) for glibenclamide–verapamil-exposed fibers in Figure8A and B. Vertical bars represent the SE of the number of fibers as indicated in Figures8. Dashed horizontal line indicates the level at which unstimulated [Ca^2+^]i was significantly greater than the prefatigue level.

As shown in Figure1, the baseline also increased during fatigue as [Ca^2+^]i failed to return to the prestimulation value before the next contraction. To evaluate this change in [Ca^2+^]i, we measured the unstimulated [Ca^2+^]i by averaging the values during the 100 msec preceding a contraction. Unstimulated [Ca^2+^]i significantly increased during the first 60 sec from 15–30 to 125–150 nmol/L for all three groups of control fibers (Fig.8A). For the control fibers that had the smallest decrease in plateau tetanic [Ca^2+^]i (in Fig.4A), unstimulated [Ca^2+^]i continued to slowly increase reaching a mean of 160 nmol/L at 180 sec (Group A). The largest decreases in unstimulated [Ca^2+^]i after the first 60 sec were observed in fibers (Group C) that had the largest reductions in plateau tetanic [Ca^2+^]i (in Fig.4C).

For the glibenclamide-exposed fibers with the smallest decrease in tetanic [Ca^2+^]i (Fig.5A), unstimulated [Ca^2+^]i rapidly increased during the first 30 sec from 24 to 65 nmol/L, increasing at a slower rate thereafter reaching a mean of 106 nmol/L by 180 sec (Fig.8B). Glibenclamide fibers that had decreases in tetanic [Ca^2+^]i exceeding 0.5 μmol/L but with no failure of Ca^2+^ release during fatigue (Fig.5B) had the largest increases in unstimulated [Ca^2+^]i reaching 203 nmol/L by 105 sec before it decreased to 112 nmol/L by 180 sec. Glibenclamide-exposed fibers that eventually failed to release Ca^2+^ upon stimulation also had significant increases in unstimulated [Ca^2+^]i, but as they failed to release Ca^2+^ unstimulated [Ca^2+^]i eventually decreased to levels that became no longer significantly different from the mean prefatigue unstimulated [Ca^2+^]i. As observed with control and glibenclamide-exposed fibers, the Kir6.2^-/-^ group of fibers with no significant decreases in tetanic [Ca^2+^]i (Fig.6A) also had rapid increases in unstimulated [Ca^2+^]i during the first 30 sec without any decrease thereafter (Fig.8C). For the group of Kir6.2^-/-^ fibers with significant decreases in tetanic [Ca^2+^]i (Fig.6B), unstimulated [Ca^2+^]i increased to 208 nmol/L by 90 sec followed by large decreases thereafter.

The proportion of fibers for which unstimulated [Ca^2+^]i increased above 200 nmol/L was 28% for control fibers and 42% for both Kir6.2^-/-^ and glibenclamide-exposed fibers, but the differences were not significant (Fischer exact test, *P* = 0.10). The highest mean value for unstimulated [Ca^2+^]i among the three groups of control fibers was 174 mmol/L compared to 208 and 203 mmol/L for Kir6.2^-/-^ and glibenclamide-exposed fibers, respectively. Finally, contrary to the study of Cifelli et al. (2007), none of the increases in unstimulated [Ca^2+^]i were large enough in Kir6.2^-/-^ or glibenclamide-exposed fibers to cause a supercontraction.

The addition of verapamil significantly reduced the extent of the increases in unstimulated [Ca^2+^]i during fatigue. First, while 52% of the glibenclamide-exposed fibers had increases in unstimulated [Ca^2+^]i exceeding 150 nmol/L, only 7% of the glibenclamide–verapamil-exposed fibers did (Fischer exact test, *P* < 0.027). Second, the highest unstimulated [Ca^2+^]i for glibenclamide–verapamil-exposed fibers was 95 nmol/L (Fig.8D) which was significantly lower (*t*-test, *P* < 0.05) than the 203 nmol/L in glibenclamide-exposed fibers (Fig.8B).

**Figure 9:**
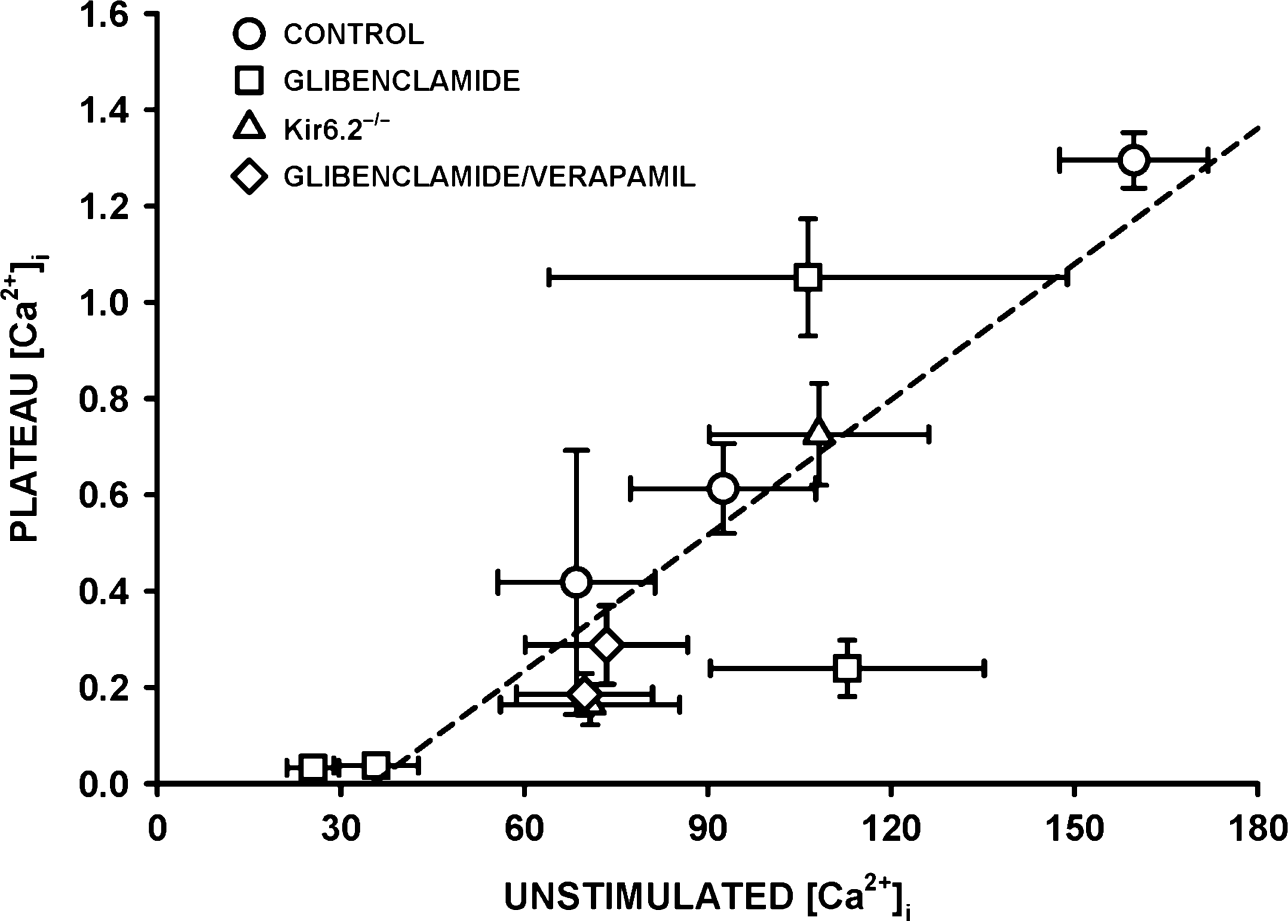
Relationship between plateau and unstimulated [Ca^2+^]i at the end of fatigue. Mean plateau [Ca^2+^]i (from data at 180 sec of each fiber group in Figs.7) were plotted against mean unstimulated [Ca^2+^]i (from the data at 180 sec of Fig.8). Dashed line is the regression line with a *R* value of 0.848.

Regardless of the experimental conditions, unstimulated [Ca^2+^]i always increased at the start of the fatigue period with only small differences between fiber groups for a given experimental condition. For fibers with no net decreases in plateau [Ca^2+^]i, unstimulated [Ca^2+^]i never decreased after the initial increases, whereas for fibers with significant decreases in plateau [Ca^2+^]i, unstimulated [Ca^2+^]i eventually decreased to different extents. To determine if the decreases in unstimulated [Ca^2+^]i were depended on how much plateau [Ca^2+^]i decreased, we plotted the mean plateau [Ca^2+^]i against the mean unstimulated [Ca^2+^]i from the data at 180 sec. The relationship appeared linear with a correlation factor of 0.848 (Fig.9). It suggests that the final unstimulated [Ca^2+^]i at 180 sec depended on the decrease in plateau [Ca^2+^]i. That is, regardless of the experimental conditions, the higher the plateau [Ca^2+^]i was at the end of fatigue (or the lower the decrease in plateau [Ca^2+^]i during fatigue) the higher was the unstimulated [Ca^2+^]i.

### Recovery period

**Figure 10:**
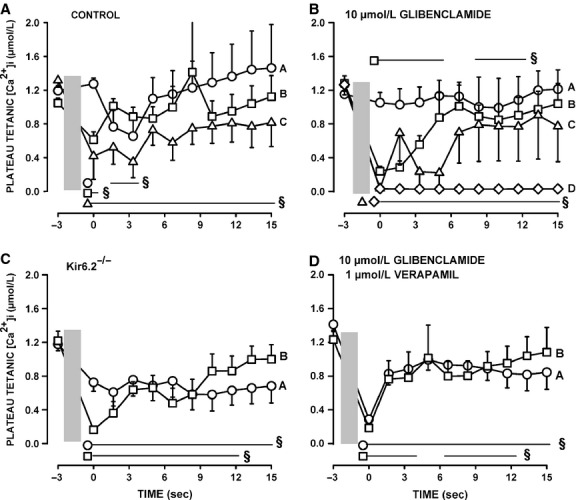
Recovery plateau tetanic [Ca^2+^]i among fiber groups. (A) Changes in plateau tetanic [Ca^2+^]i following fatigue for the three control groups as shown in Figure5A–C; (B) the same for glibenclamide-exposed fibers as shown in Figure6A–D; (C) for Kir6.2^-/-^ fibers in Figure7A–C; (D) for glibenclamide- and verapamil-exposed fibers as shown in Figures8A–B. During the recovery period, fibers were stimulated every 100 sec. Gray vertical box represents the 3 min fatigue period. Vertical bars represent the SE of the number of fibers as indicated in Figures8. ^§^Mean plateau tetanic [Ca^2+^]i was significantly different from the prefatigue mean value (Time −3 min). ANOVA and L.S.D., *P* < 0.05.

Among the three groups of control fibers, only the one with the largest decreases in plateau tetanic [Ca^2+^]i failed to fully recover after 15 min of recovery (Fig.10A). All glibenclamide-exposed fibers with decreases in plateau tetanic [Ca^2+^]i exceeding 0.5 μmol/L had very slow recovery (Fig.10B). Fibers with a complete failure of Ca^2+^ release by 100 sec during fatigue completely failed to recover their capacity to release Ca^2+^, whereas among the fibers which failed to release Ca^2+^ by 150 sec some were able to recover while others completely failed giving rise to large SE for this group. Both groups of Kir6.2^-/-^ fibers also had very slow recovery (Fig.10C). Finally, while glibenclamide and verapamil fibers also had incomplete recovery of plateau tetanic [Ca^2+^]i, they reached a new steady state within 100 sec (Fig.10D) which was much shorter than for those exposed to just glibenclamide.

## Discussion

This study reports for the first time at 37°C: (1) large differences in fatigue kinetics among skeletal muscle fibers; (2) differences in fatigue kinetics between Kir6.2^-/-^ and glibenclamide-exposed muscle fibers; (3) that not all fibers were adversely affected by a lack of KATP channel deficiency; and (4) that verapamil affected both unstimulated and tetanic [Ca^2+^]i.

### Differences in fatigue kinetics among fiber types

Many studies on muscle fatigue have been carried out at 22°C using single FDB fibers by the Allen/Westerblad groups (e.g., Westerblad and Allen 1991, 1992; Duty and Allen 1995; Chin and Allen 1996, 1997, 1998; Chin et al. 1997; Andrade et al. 1998), and none of them mentioned differences in kinetics between fibers. This is most likely because in an earlier study, Lännergren and Westerblad (1991) reported that 73% of 26 fibers lost 50% of their force during fatigue between 4 and 7 min; only 4% of the fibers had a 50% force loss in 2 min and another 12% with a time exceeding 12 min. This is rather surprising considering that the major fiber types in FDB are I-IIA (6%), I-IIX (8%), IIA (19%); IIA-IIX (32%); IIX (21%) (Banas et al. 2011). In this study, in which fatigue was elicited at 37°C, the fatigue kinetics of control fibers were divided into three distinct groups with clear differences in the decrease in tetanic [Ca^2+^]i (Figs.3, 4). The first group comprised 28% of the 18 tested fibers, had at the end of the fatigue period greater mean tetanic [Ca^2+^]i than the prefatigue mean and are expected to be the most oxidative fibers, possibly type I-IIA, I-IIX, and IIA. The second group or 44% of the fibers had a net decrease in tetanic [Ca^2+^]i varying between 0.1 and 0.5 μmol/L, and may be the type IIA-IIX fibers. The third and last group with 28% of the fibers had decreases exceeding 0.5 μmol/L and is likely to comprise the most glycolytic FDB fibers type IIX fibers).

A final difference is the occurrence of fade, which was observed when fibers failed to maintain a Ca^2+^ plateau phase during contractions, a situation that has never been reported in the Allen/Westerblad studies. One mechanism causing fade is a failure of a fiber to generate an action potential to all electrical stimuli as reported by Cairns et al. (2003) when soleus muscles are exposed to 40 mmol/L Na^+^ and stimulated at 125 Hz. It is unlikely that a failure to generate some action potentials upon stimulation during fatigue is due to a stimulus strength that becomes sub-threshold for two reasons. First, our electrical pulse stimulation is set at a voltage that is 2.5 times above the threshold measured prior to fatigue. We are also careful at setting the pulse duration to 0.3 ms because longer pulse duration, especially at high voltages, can directly stimulate Ca^2+^ release in the presence of tetrodotoxin (TTX), a Na^+^ channel inhibitor, to prevent action potentials because long pulses eventually depolarized t-tubular membrane activating CaV1.1 channels (Cairns et al. 2007). Second, in our past studies an increase in voltage has never caused an increase in force or disappearance of fade. So, the most likely mechanism for a failure to generate action potentials to all stimuli is most likely related to the concomitant activation of KATP and ClC-1 Cl^−^ channels; an activation that occurs during metabolic stress (Pedersen et al. 2009a,b).

There are two differences between this study and those of Allen/Westerblad. The first one is the stimulation protocol: here we used one 200 msec contraction every sec for 3 min, whereas they used 400 msec tetanic contractions every 4 sec then every 3, 2, 1, and 0.5 sec with changes in time interval every min until force decreases by 40% or 50%. Fatigue is a phenomenon that is triggered by an energy deficit to prevent large ATP loss that can cause fiber damage or cell death (McKenna et al. 2008; Pedersen et al. 2009a,b; MacIntosh et al. 2012). Thus, regardless of the stimulation protocol, the more oxidative fibers should have slower decay in tetanic [Ca^2+^]i and force than the more glycolytic fibers because the former are more efficient in generating ATP.

The second and most important difference between the studies is the experimental temperature; that is, 22°C versus 37°C, the latter being physiological because muscle temperature in vivo easily exceeds 37°C during exercise (Brooks et al. 1971). The activity of ion transporters, such as the Na^+^ K^+^ ATPase pump, is greater while the depressive effects of the increases in extracellular K^+^, intracellular inorganic phosphate and H^+^ on force are much less at 30–37°C than at 20–25°C (Westerblad et al. 1997; Pedersen et al. 2003; Debold et al. 2006). So, the proportion of the force decrease during fatigue related to changes in inorganic phosphate, pH, Na^+^, and K^+^ is very likely to be greater at 22°C than at 37°C. Furthermore, at 22°C, glibenclamide only produces a partial reversal of fatigue and in just 50% of the tested FDB fibers (Duty and Allen 1995). The extent of the partial reversal is similar to that reported in tibialis muscle subjected to low-intensity workload at 30°C that only causes small decreases in twitch force (Zhu et al. 2014). At 37°C, contractile dysfunctions and fiber damage are observed during intense muscle activity leading to fatigue in KATP-channel-deficient muscle in vivo and in vitro at 37°C (Gong et al. 2000, 2003; Matar et al. 2000; Thabet et al. 2005; Cifelli et al. 2007, 2008). Considering that the extent of the KATP channel activation increases with the extent of metabolic stress, then the lack of any contractile dysfunctions during fatigue at 22°C in the presence of glibenclamide is very likely due to a lower metabolic stress and thus less KATP channel activation at 22°C than at 37°C; the lower metabolic stress at 22°C being related to greater Na^+^ and K^+^ depression on membrane excitability causing greater decrease in tetanic [Ca^2+^]i and force – that is, lower ATP demand by Ca^2+^ ATPase and myosin ATPase. Finally, mitochondrial respiration and ATP production increases more than twofold from 25°C to 37°C (Dufour et al. 1996) and thus one can expect greater differences in fatigue kinetics between oxidative and glycolytic fibers at 37°C. We therefore suggest that the greater variability in fatigue kinetics at 37°C than at 22°C among FDB fibers is primarily because of greater metabolic demand and greater capacity of oxidative fibers to generate ATP. This study is thus another example as to why we must study the etiology of muscle fatigue at the physiological temperature of 37°C.

### Myoprotective role of the KATP channel activity

#### Importance of KATP channel among fibers

Studies using KATP channel agonist have provided evidence that the channel contributes to a decrease in force by reducing action potential amplitude and the amount of Ca^2+^ released by the sarcoplasmic reticulum (Duty and Allen 1995; Matar et al. 2000; Gong et al. 2003; Zhu et al. 2014). While the absence of KATP channel activity during low-intensity workload only prevents the small reduction in action potential amplitude and force observed in control muscles (Zhu et al. 2014), during intense muscle activity leading to fatigue the absence of channel activity causes fiber damage (Thabet et al. 2005) and contractile dysfunctions (Cifelli et al. 2007, 2008; Boudreault et al. 2010), including large membrane depolarization, large increases in unstimulated [Ca^2+^]i and force resulting in apparent faster fatigue rate and lower capacity to recover following fatigue. Here, we again showed that compared to control fibers the absence of KATP channel activity resulted in significantly greater proportions of fibers with (1) decreases in tetanic [Ca^2+^]i exceeding 0.5 μmol/L, (2) fade, and (3) complete failure to release Ca^2+^ upon stimulation (the latter observed with glibenclamide-exposed fibers). So, as previously suggested, KATP channels play an important myoprotective role during muscle activity, especially during fatigue.

One group of glibenclamide-exposed fibers had peak tetanic [Ca^2+^]i that remained above prefatigue level while plateau tetanic [Ca^2+^]i decreased slightly but not significantly (Fig.5A). The fatigue kinetics of this group of fibers resembled those of the most fatigue-resistant fibers in control conditions (Fig.4A). There was also one Kir6.2^-/-^ fiber for which neither peak nor plateau tetanic [Ca^2+^]i became less than the prefatigue level (Fig.3E and F). Stimulating fibers with 3.5 sec train of action potentials every 7 sec eventually causes after 2000 action potentials the activation of KATP and ClC-1 channels in glycolytic EDL fibers but not in oxidative soleus fibers even after 15,000 action potentials (Pedersen et al. 2009a,b). Thus, the KATP channel deficient fibers that had similar fatigue kinetics to those of the most fatigue-resistant control fibers are most likely the oxidative fibers type I-IIA and I-IIX that are capable of generating sufficient ATP to prevent any KATP channel activation in normal fibers so the addition of glibenclamide is without effect. For the remaining three groups of glibenclamide-exposed fibers the decreases in tetanic [Ca^2+^]i exceeded 0.5 μmol/L, with one group able to release Ca^2+^ throughout the entire fatigue period while a second group stopped releasing Ca^2+^ near 150 sec and the third group near 100 sec suggesting that different fibers have different degrees of dependency on KATP channels. Taken into account that the t-tubular KATP channel protein content is in the order of IIB > IIX > IIA > I (Banas et al. 2011), we suggest that the severity of the decreases in tetanic [Ca^2+^]i in the three affected glibenclamide-exposed fibers observed was in the order of type IIX > IIA-IIX > IIA fibers.

#### Differences between the two KATP-channel-deficient models

Previous studies on the effect of KATP channel deficiency have reported no difference in fatigue kinetics between Kir6.2^-/-^ and glibenclamide-exposed mouse EDL and soleus muscle (Gong et al. 2000, 2003). With smaller muscle preparations, that is, FDB bundles and single fibers (Cifelli et al. 2007), small differences between Kir6.2^-/-^ and glibenclamide-exposed wild-type fibers were observed, as well as some glibenclamide effects in Kir6.2^-/-^ fibers, the most obvious being a significantly greater reduction in force recovery following fatigue in the presence than in the absence of glibenclamide. Here, we now report more differences between Kir6.2^-/-^ and glibenclamide-exposed fibers. First, although not significant, glibenclamide-exposed fibers had a tendency for greater decreases in plateau tetanic [Ca^2+^]i than Kir6.2^-/-^ fibers. Second, the proportion of fibers with decreases in peak tetanic [Ca^2+^]i exceeding 0.5 μmol/L was not significantly different between control and Kir6.2^-/-^ fibers, whereas it was significantly greater in glibenclamide-exposed fibers than in both control and Kir6.2^-/-^ fibers. Third, compared to control, the proportion of fibers that completely failed to release Ca^2+^ upon stimulation was significantly higher in glibenclamide-exposed fibers but not in Kir6.2^-/-^ fibers (Fig.2). Fourth, tetanic [Ca^2+^]i of all Kir6.2^-/-^ fibers returned toward prefatigue levels during recovery while many glibenclamide-exposed fibers failed to recover. So, abolishing KATP channel activity with glibenclamide caused more contractile dysfunctions when compared with the Kir6.2^-/-^ knockout model.

There is no evidence that glibenclamide affects other channels than the KATP channel in unfatigued skeletal muscle (Light and French 1994; Barrett-Jolley and McPherson 1998). Mammalian skeletal muscle including FDB fibers have large Cl^−^ conductance (Geukes Foppen 2004; Pedersen et al. 2009a) and exposing FDB fibers to glibenclamide has no effect on membrane conductance (E. Hesse and J. M. Renaud, unpubl. data). However, when ClC-1 Cl^−^ and KATP channels are concomitantly activated in EDL fibers during metabolic stress (Pedersen et al. 2009a), the sum of the individual reduction in membrane conductance by glibenclamide and 9-AC, a ClC-1 Cl^−^ channel blocker, exceeds the total increase in membrane conductance. The mechanism of action of glibenclamide involves binding to SUR2A, which then closes the Kir6.2 pore (Gribble and Reimann 2002). SUR2A and the cystic fibrosis transmembrane conductance regulator (CFTR), a Cl^−^ channel, are both member of the ATP-binding–cassette (ABC) protein family (Higgins 1995). When activated, CFTR reduces the activity of epithelial Na^+^ channels in lung tissue (Mall et al. 1998; Lueck et al. 2010). In pancreatic β-cells, SUR1 interacts with the L-type Ca^2+^ channels (Shibasaki et al. 2004) whereas SUR2A form complexes with glycolytic enzymes (Dhar-Chowdhury et al. 2005). Considering that ClC-1 and KATP channels are concomitantly activated during metabolic stress, it will be interesting in future studies to determine whether SUR2A modulates the ClC-1 channel activity and whether glibenclamide blocks this modulation. If it is the case, then a glibenclamide inhibition of both KATP and ClC-1 channel activity in wild-type fibers would be expected to cause more contractile dysfunctions than just a loss of KATP channel activity in Kir6.2^-/-^ fibers.

Another possibility to explain the differences between Kir6.2^-/-^ and glibenclamide-exposed fibers is the fact that Kir6.2^-/-^ represents a chronic loss of KATP channel activity that allows for compensatory mechanisms to develop. In fact, daily O2 uptake is greater in Kir6.2^-/-^ than in wild-type mice (Alekseev et al. 2010; Scott 2012) and mitochondrial glucose oxidation increases within 60 sec during fatigue in Kir6.2^-/-^ FDB bundles and only during recovery in wild-type bundles (Scott 2012). So, one compensatory mechanism may be a greater ATP production associated with greater oxidative capacity that reduces the dependency of Kir6.2^-/-^ fibers on the myoprotection of KATP channels.

The remaining question is why no difference between the two KATP-channel-deficient models had not been reported in previous studies? The lack of difference with EDL and soleus is most likely because of the presence of a significant anoxic core during fatigue as per Barclays’ model (Barclay 2005) preventing any substantial glucose oxidation to take effect in Kir6.2^-/-^ muscles. However, this study is reporting greater differences between glibenclamide and Kir6.2^-/-^ single FDB single fibers than the study of Cifelli et al. (2007). In this study, FBS was added to the physiological solution and experiments were carried out the day fibers were prepared whereas Cifelli et al. did not used FBS and experiments were carried out the day after the preparation. FBS improves single fiber viability and stability especially when stressed with fatigue. Furthermore, single fibers are stable for 3 h in the experimental chamber when used the day of the preparation while they are not the next day (Selvin et al. 2014). It is thus possible that the level of physiological stress of single FDB fibers was higher in the Cifelli et al. study diminishing the capacity of compensatory mechanisms in Kir6.2^-/-^ fibers to prevent large contractile dysfunctions. This notion is further supported by the fact that Cifelli et al. reported that 65% of the KATP-channel-deficient fibers supercontracted, an effect not observed in this study.

### THE importance of CaV1.1 channels in the increase in unstimulated [Ca^2+^]_i_ in glibenclamide-exposed fibers

In this study, fibers were loaded with fura-2 by exposing them to fura-2 AM, which can permeate membrane organelles before esterases cleave the AM. Consequently, not all of the Ca^2+^-fura-2 fluorescence may come from cytosolic Ca^2+^, an important point to consider for unstimulated [Ca^2+^]i. Unstimulated [Ca^2+^]i of unfatigued fiber was still above resting level one sec after a contraction (e.g., 1st contraction, Fig.1A), a situation that has also been reported when fura-2 or indo-1 are micro-injected in fibers (Westerblad and Allen 1991; Chin and Allen 1996, 1998; Chin et al. 1997). For control fibers, mean unstimulated [Ca^2+^]i increased to values approaching 180 nmol/L (Fig.8A), which is also similar to the mean value of 177 nmol/L previously reported with microinjected indo-1 (Chin et al. 1997). It is thus unlikely that noncytosolic fura-2 fluorescence substantially contributed to unstimulated [Ca^2+^]i.

Unstimulated [Ca^2+^]i significantly increased in control, glibenclamide-exposed and Kir6.2^-/-^ fibers, mostly between the 60th and 90th sec. The subsequent changes in unstimulated [Ca^2+^]i appeared to depend on the fate of the plateau [Ca^2+^]i. That is, decreases in unstimulated [Ca^2+^]i occurred only in fibers with decreases in plateau [Ca^2+^]i; the decreases in unstimulated [Ca^2+^]i being the largest in fibers with the largest decreases in plateau [Ca^2+^]i giving rise to a linear relationship between the two parameters (Fig.9). This situation most likely arises because as the amount of Ca^2+^ release during contraction diminishes, Ca^2+^ pumps are more capable of lowering [Ca^2+^]i between contractions.

Cifelli et al. (2008) reported greater unstimulated force and membrane depolarization to -30 mV in KATP channel deficient than in control FDB. Then, using verapamil, they provided indirect evidence that the depolarization was large enough to activate CaV1.1 channels allowing for large Ca^2+^ influx between contractions that subsequently caused large unstimulated force. As expected, verapamil significantly lowered unstimulated [Ca^2+^]i in glibenclamide-exposed fibers, but the effect appears not limited to a reduction in Ca^2+^ influx between contractions. This is because verapamil also caused significant reductions in tetanic [Ca^2+^]i primarily because of substantial fade, an effect also observed for tetanic force (Cifelli et al. 2008). In fact, the data points for the plateau versus unstimulated [Ca^2+^]i from the glibenclamide- and verapamil-exposed fibers fell closed to the relationship from the other experimental conditions (Fig.9). Thus, the prevention of large increases of unstimulated force reported earlier in KATP-channel-deficient muscles (Cifelli et al. 2008) is the result of a reduced Ca^2+^ load due to lower Ca^2+^ influx through CaV1.1 channels between and during contractions. Another verapamil effect was a significant improvement to recover tetanic [Ca^2+^]i in glibenclamide-exposed fibers (Fig.10B and D). This is in agreement with the fact that verapamil improved force recovery in glibenclamide-exposed FDB bundles (Cifelli et al. 2008). Thus, this study strengthens our previous conclusion that the large increase in unstimulated [Ca^2+^]i in KATP-channel-deficient fibers causes fiber damage resulting in lower capacity to recover.

In conclusion, this study demonstrated large differences in fatigue kinetics among FDB fibers during fatigue at 37°C, differences that are much greater than those reported in previous studies carried out at 22°C. Abolishing KATP channel activity using pharmacological or genetic approach causes contractile dysfunctions in most but not all FDB fibers; it is proposed that oxidative fibers are the least dependent and glycolytic the most dependent on the myoprotection from KATP channels. This study shows for the first time that Kir6.2^-/-^ fibers develop less contractile dysfunctions than glibenclamide-exposed wild-type fibers and it is suggested that the greater the physiological or metabolic stress the smaller the differences between the two KATP channel deficiency models. Finally, this study provides further evidence that a large Ca^2+^ influx through CaV1.1 channels between and during contractions in KATP-channel-deficient fibers causes a Ca^2+^ load resulting in large increases in unstimulated [Ca^2+^]i and force resulting in lower capacity to recover, possibly due to some fiber damage.

## Conflict of Interest

None declared.
